# Current and Emerging Insights into the Causes, Immunopathogenesis, and Treatment of Cutaneous Squamous Cell Carcinoma

**DOI:** 10.3390/cancers17101702

**Published:** 2025-05-19

**Authors:** Ronald Anderson, Nomzamo M. Mkhize, Mahlatse M. C. Kgokolo, Helen C. Steel, Theresa M. Rossouw, Lindsay Anderson, Bernardo L. Rapoport

**Affiliations:** 1 The Clinical and Translational Research Unit, The Medical Oncology Centre of Rosebank, Saxonwold, Johannesburg 2196, Gauteng, South Africa; ronald.anderson@rosebankoncology-ctru.co.za; 2 Department of Dermatology, Faculty of Health Sciences, University of Pretoria, Prinshof, Pretoria 0084, Gauteng, South Africa; nomzamomkhize9@gmail.com (N.M.M.); mahlatse.kgokolo@up.ac.za (M.M.C.K.); 3 Department of Immunology, Faculty of Health Sciences, University of Pretoria, Prinshof, Pretoria 0084, Gauteng, South Africa; helen.steel@up.ac.za (H.C.S.); theresa.rossouw@up.ac.za (T.M.R.); 4 Curo Oncology, Les Marais, Pretoria 0084, Gauteng, South Africa; curo3onc@curo-oncology.co.za

**Keywords:** adenosine, co-inhibitory immune checkpoints, cutaneous squamous cell carcinoma, driver-mutations, human papillomavirus, non-coding RNAs, prostaglandin E2, regulatory T cells, transforming growth factor-β1, ultraviolet radiation

## Abstract

The increasing incidence of cutaneous squamous cell carcinoma (cSCC), together with the ominous risks of metastasis and recurrence, underscores the importance of identifying novel therapies and validated biomarkers to augment patient management, particularly in the context of well-established and advanced disease. Following a brief overview of the well-recognized epidemiology, clinical features, and diagnosis of cSCC, the current review is focused on risk factors, most prominently excessive exposure to ultraviolet radiation (UVR) as a cause of persistent, pro-tumorigenic mutagenesis, and immune suppression. The next phase of the review encompasses an evaluation of the search for key driver mutations in the pathogenesis of cSCC, including the role of these and other mutations in the formation of immunologically reactive neoepitopes. With respect to additional mechanisms of tumorigenesis, immune evasion is prioritized, specifically the involvement of cell-free and infiltrating cellular mediators of immune suppression. Prominent amongst the former are the cytokine, transforming growth factor-β1 (TGF-β1), the prostanoid, prostaglandin E2, and the emerging immune suppressive nucleoside adenosine. In the case of the latter, tumor-infiltrating and circulating regulatory T cells have been implicated as being key players. The final sections of the review are focused on an update of the immunotherapy of established and advanced disease, as well as on the search for novel, reliable lesional and systemic biomarkers with the potential to guide patient management.

## Simple Summary

The incidence of cutaneous squamous cell carcinoma is increasing worldwide coincident with global warming. The high mutational burden and associated immunosuppression are the most prominent triggers. Once established, persistence of the tumor is achieved via localized and systemic mechanisms of immune evasion. Prominent mediators of immune evasion include transforming growth factor-β1 (TGF-β1), prostaglandin E2 (PGE2) and regulatory T cells. Circulating tumor DNA represents a promising systemic biomarker of disease progression. Combined targeting of TGF-β1, PGE2 and PD-1 shows considerable therapeutic promise.

## 1. Introduction

Cutaneous squamous cell carcinoma (cSCC), which develops from pre-malignant actinic keratosis lesions, is the second most common type of non-melanoma skin cancer. It is a predominantly localized, indolent tumor, which is amenable to surgical excision, often without the necessity for adjunctive chemotherapy, targeted therapy, immunotherapy, or radiation therapy [1]. Due to the availability and efficacy of local treatments, together with the potentially protective early effect of a high tumor mutational burden [1,2,3,4], there has been little incentive to probe key mechanisms involved in the immunopathogenesis of cSCC, which, consequently, have been somewhat under-explored [4]. This situation is, however, changing with the recognition of a steadily increasing incidence rate of cSCC worldwide, largely due to the ominous threat posed by global warming, resulting in intensified exposure of keratinocytes to the damaging, pro-tumorigenic levels of solar ultraviolet (UV)A (UVA) and UVB radiation [5].

The initial section of this review covers the epidemiology, clinical features, and diagnosis of cSCC, albeit briefly, given the publication of several recent comprehensive reviews on these topics. This is followed by a more extensive coverage of risk factors, including, but not limited to, the primary roles of cumulative exposure to UV radiation (UVR) and immunosuppression, as well as the involvement of smoking and infection caused by human papillomavirus (HPV) and human immunodeficiency virus (HIV) and tattooing as additional contributors to tumorigenesis. The subsequent sections of the review are focused on the profile of potential driver mutations in cSCC and their link to neoantigen expression, as well as current and novel mechanisms of immune evasion, including intra-lesional and systemic mechanisms, which drive the progression of this skin malignancy. The final sections address the potential of these mechanisms of immune evasion to serve as targets for the development of innovative immune-based therapies, as well as the identification of novel cellular and systemic biomarkers with predictive/prognostic promise, these being key areas for future research.

## 2. Epidemiology, Clinical Features, and Diagnosis of Cutaneous Squamous Cell Carcinoma (cSCC)

### 2.1. Epidemiology

As mentioned above, cSCC is the second most common non-melanoma skin cancer (NMSC), following basal cell carcinoma (BCC), and accounts for approximately 20% of all skin malignancies [6]. The incidence of cSCC has been rising globally, largely due to increasing UVR exposure, aging populations, and improved detection rates [2]. Chronic sun exposure remains the most significant risk factor, particularly in fair-skinned individuals with Fitzpatrick skin types I and II. Other contributing factors include immunosuppression, chronic inflammation, genetic predisposition, and environmental exposures such as arsenic and industrial chemicals [3].

Multicentric cSCCs occur in an estimated 2.1% of subjects within the same year of diagnosis. Dermoscopy can enhance the early detection of cSCC compared to visual inspection. However, its use does not improve disease outcome. Accordingly, a comprehensive clinical assessment is also essential to guide the proper work-up and management of cSCC [7].

### 2.2. Clinical Features

Cutaneous SCC commonly presents as scaly, erythematous, tender, or painful hyperkeratotic plaques, papules, or nodules, often with central ulceration or crusting. Lesions may arise de novo or from pre-malignant lesions such as actinic keratoses or Bowen’s disease (in situ SCC) [2]. The tumor frequently appears on sun-exposed areas, including the face, scalp, ears, neck, and dorsal hands, though it can also occur in less-exposed areas, particularly in immunosuppressed patients and elderly males [2].

While many cSCCs grow slowly, certain high-risk features indicate a more aggressive phenotype and growth rate. These include the following [8,9]:

• Tumor diameter > 2 cm;
• Depth of invasion > 6 mm;
• Poor differentiation;
• Perineural invasion (PNI);
• Location on high-risk sites (e.g., ears, lips, or genitals);
• Immunosuppression or a prior history of aggressive SCC [3].

Locally advanced cSCC can invade deeper structures, including muscle, bone, and nerves, leading to significant morbidity. Metastasis, while relatively rare (occurring in about 2–5% of cases), is more common in high-risk tumors, with regional lymph nodes being the most frequent site of spread [4].

### 2.3. Diagnosis

The diagnosis of cSCC is primarily clinical, supported by dermoscopy, and confirmed through histopathological examination following biopsy. However, it is important to consider that the early forms of cSCC may resemble BCC or inflammatory diseases, posing a clinical diagnostic challenge. In ulcerated forms, the presence of ulceration and blood spots may conceal and modify the dermoscopic characteristics of the lesion, complicating the diagnosis [10]. Early detection remains critical, particularly in high-risk populations [10]. Innovative new technologies such as reflective confocal microscopy (RCM), optical coherence tomography (OCT), and fluorescence molecular imaging (FMI) are showing promise in the diagnosis and identification of dermatological malignancies [11,12,13,14]. These procedures “offer benefits including non-invasiveness, rapidity, comprehensive lesion imaging, and enhanced sensitivity, but face challenges like high costs and the need for specialized expertise” [15]. Additional issues include the fact that these sophisticated procedures are costly and not widely available and/or reimbursed.

#### 2.3.1. Clinical and Dermoscopy Evaluation

Dermoscopy remains an important aid in distinguishing cSCC from other cutaneous lesions. Key dermoscopy features include the following:

• Scaling and white structureless areas;
• Dotted or glomerular vessels;
• Ulceration in more advanced tumors [2].

#### 2.3.2. Histopathology and Grading

A definitive diagnosis requires a biopsy, with either shave, punch, or excisional techniques, depending on the size and location of the lesion. Histopathologically, cSCC is characterized by atypical keratinocytes extending beyond the epidermis into the dermis. Tumor grading (well, moderately, or poorly differentiated) is based on cellular atypia, degree of keratinization, and mitotic activity [1].

Early recognition and accurate diagnosis of cSCC are critical for effective management, particularly in patients with high-risk features or immunosuppression. With the rising incidence of cSCC, continued advances in diagnostics and treatment strategies are essential to improving patient outcomes.

The Brigham and Women’s Hospital (BWH) system, the Union for International Cancer Control eighth edition (UICC), and the American Joint Committee on Cancer (AJCC) staging eighth edition are among the staging systems used for the classification of cSCC [8,16]. Staging can help identify patients who require further work-up and additional treatment, including surgery, adjuvant radiation, and/or systemic therapy [8,16].

## 3. Risk Factors

### 3.1. Ultraviolet Radiation

Direct evidence for the induction of non-melanoma skin malignancies and underlying DNA damage has been derived from experiments in mice and rats, while an abundance of epidemiological data has confirmed that this association also holds for human skin [17,18].

Progressive exposure of the skin to the damaging and immunosuppressive effects of UVR, be it natural (sunlight predominantly) or artificial (e.g., tanning beds), is the key risk for the development of cSCC, augmented by pre-existing malignancy, iatrogenic immunosuppression, infection, genetic disorders such as Fanconi anemia, Bloom syndrome, ataxia telangiectasia, and others, aspects of lifestyle, gender, climatic influences, and geographic location [1,2,3,4,5,19,20]. The exposure of keratinocytes to UVR poses the risk of oxidative damage and other types of chemical alteration to the bases of DNA, such as the formation of photodimers, especially cyclobutane pyrimidine dimers and pyrimidine-6,4-pyrimidinone dimers [4,21,22]. If not repaired by various types of DNA repair enzymes, especially those that promote nucleotide excision repair, the efficiency of which declines with advancing age, the resultant impairment of genomic integrity predisposes to mutagenesis and the development of cancer [21,22]. In addition to these pro-carcinogenic activities, UVR, as mentioned earlier, also drives localized inflammation and associated immunosuppression, which also contribute to skin carcinogenesis [19]. Notwithstanding the production of DNA-damaging reactive oxygen species (ROS) and nitrogen species (RNS) by skin phagocytes, additional mediators of inflammation and immunosuppression derived from UVR-exposed keratinocytes, Langerhans cells, mast cells, and nerve cells, include cytokines, interleukin (IL)-4, IL-10, and transforming growth factor-β1 (TGF-β1), all of which promote the recruitment and activation of regulatory CD4^+^ T cells (Tregs), as well as the broadly immunosuppressive prostanoid prostaglandin E2 (PGE2) [19,23,24].

### 3.2. Radiation Therapy

Radiation therapy (RT) is a cornerstone in the treatment of various cancers, including cSCC, which may pose the potential risk of mutagenesis. However, in this setting, the benefits clearly outweigh potential risks, particularly when surgical options are limited or when aiming to improve local control of the disease. For example, adjuvant radiotherapy has been associated with improved disease-free survival (DFS) and overall survival (OS) in patients with high-risk cSCC, especially those with features that include PNI or regional lymph node involvement [25]. Nevertheless, RT has been associated with an increased risk of developing certain types of skin cancer.

In this context, evidence indicates that exposure to RT is linked to a higher incidence of BCC. However, the relationship between therapeutic ionizing radiation and cSCC is less clear. An increased risk for both BCC and cSCC in the field of radiation exposure has been described in a study published in the Archives of Dermatology [26]. Conversely, another study suggested that exposure to RT is associated with the risk of BCC, but not cSCC [27]. Given these conflicting results, it is important to recognize that while RT may increase the risk of the development of skin cancers, the risk is relatively low and, as mentioned above, is outweighed by the significant benefits in disease control and improvement in OS. Importantly, the risk of developing radiation-induced SCC is relatively low, with studies indicating a 10-year probability of developing a radiation-induced cancer to be approximately 0.8% in certain patient groups [28]. Nevertheless, patients with documented pre-existing risk factors and their attending physicians should be particularly vigilant.

### 3.3. Iatrogenic Immunosuppression

Recipients of solid organ transplants represent a group at particularly high risk for the development of aggressive cSCC [4,29,30]. In this setting, these patients experience rates of occurrence, which are not only approximately 100-fold greater than those associated with seemingly healthy persons and other immune-suppressed groups, but also with higher numbers of skin tumors, as well as higher rates of recurrence and risk of metastasis [4,29,30]. Concerningly, immunotherapy is not a preferred option in these patients due to the attenuation of the efficacy of anti-rejection chemotherapy [30]. Although the risk is lower than in those with solid cancers, increased vigilance is also necessary in those receiving immunosuppressive medications for non-malignant conditions, as well as those with acquired and inherited immune deficiency disorders.

### 3.4. Smoking

While the association of smoking with the development and progression of head and neck SCC (HNSCC) is well-recognized [31,32], the relationship of smoking with the development of cSCC is, however, somewhat contentious, with a number of conflicting studies reported in the medical literature. In this context, an earlier study reported by De Hertog et al. described a Dutch hospital-based case–control study undertaken to identify possible relationships between smoking and the occurrence of different types of dermatological malignancy. These were cSCC (n = 161), nodular BCC (NBCC, n = 301), superficial multifocal BCC, and malignant melanoma (n = 125), as well as 386 control participants [33]. The authors reported a statistically significant overall association of smoking history with cSCC [relative risk (RR) = 2.3; *p* < 0.0001], which was highest for current smokers (RR = 3.3), decreasing to 1.9 for former smokers [31]. Following adjustment for age, sex, and sun exposure, the RR remained significant at 2.0 (*p* = 0.008). A dose/response relationship was evident with the number of cigarettes smoked/day in the group of cSCC participants. However, no associations between smoking history and the other three types of skin cancer were evident. The authors concluded that “tobacco smoking is an independent risk factor for cutaneous squamous cell carcinoma” [33].

In a later study, Arafa et al. performed a meta-analysis of cohort studies focused on smoking history and the risk of three different types of skin cancer, namely, cSCC, BCC, and malignant melanoma [34]. The analysis comprised 15 suitable studies, of which 6, 6, and 8 encompassed one or more of cSCC, BCC, and malignant melanoma, respectively. The authors reported a statistically significant pooled RR value of 1.32 [95% confidence intervals (CIs) 1.15, 1.52] for the association of smoking history with cSCC, which was strongest with heavy smoking. No meaningful risks were associated between BCC or melanoma with smoking history. Although the association was moderate, the authors concluded that “current smoking and heavy smoking were associated with a higher risk of SCC” [34].

Taking an alternative approach to minimize the limitations of dependence on the accuracy of smoking histories, Lee et al. applied an analysis of genetic determinants apparently linked to smoking initiation, amount smoked, and lifetime smoking exposure to investigate associations between smoking and cSCC [35,36,37]. Using a two-sample Mendelian randomization/genome-wide association (GWAS) study design, the authors analyzed GWAS summary statistics from the “Kaiser Permanente” Resource for Genetic Epidemiology Research on Aging (GERA) [35]. “Kaiser Permanente” is a California-based, integrated managed care consortium. The study cohort analyzed by Lee et al. comprised 7701 cases of cSCC and 60167 controls (all white non-Hispanics). The authors found “modest evidence” indicative of an association of lifetime smoking with cSCC [odds ratio (OR) = 1.47, *p* = 0.012]. However, no associations were evident between either genetically associated correlates of smoking initiation or amount smoked and risk for cSCC. The authors concluded that lifetime smoking may be “a causal risk factor for cSCC”, while emphasizing the urgency of avoidance of the smoking habit, as well as highlighting the possible limitations of their study [35]. The latter included the heterogeneous nature of their study population, the possible role of epigenetic changes that could affect gene expression, and the inherent limitations of Mendelian randomization in “determining causal associations” [35].

In an even more recent Finnish hospital-based, cross-sectional study, spanning the period May 2017–October 2020, a total of 488 participants (242 females and 246 males aged 21–79 years) was investigated by Uotila et al. for the presence of various types of skin lesions, including cSCC and possible associations with tobacco smoking [38]. The authors reported a significant association of smoking with cSCC when comparing ever-smokers with non-smokers (OR = 1.99, *p* = 0.043). However, no associations with BCC or melanoma were evident [38].

Although for the balance of evidence, the aforementioned studies appear to support a relationship between smoking and the occurrence of cSCC, the strength of the association is nevertheless debatable and has not been detected in several studies. In one of these, a nationwide large cohort study undertaken in Sweden in 1971–2000, Odenbro and colleagues investigated the association of smoking and snuff use with the occurrence of cSCC in male construction workers (n = 337,311) [39]. During the 30-year follow-up period, “a total of 756 incident cases of cSCC occurred”. However, no significant association was found between smoking and risk of cSCC, leading the authors to conclude that tobacco use does not increase the risk of cSCC. On a cautionary note, however, the geographic location of this study and the probable use of protective, safety gear by construction workers represent potential caveats.

A second negative study, which had a 16-year longitudinal population-based design, was undertaken during 1992–2007 in a sub-tropical region of Eastern Australia by McBride et al., who investigated the association between tobacco smoking and the risk of cSCC [40]. The authors recruited eligible adults (n = 1287, aged 25–75 years, of whom 43% were male) who had no prior history of cSCC. Neither current nor former smokers were found to be at risk for the development of cSCC, with respective RR values of 1.5 and 1.1 [40]. Furthermore, no dose/response relationships or associations with duration of smoking were evident. Accordingly, the authors concluded that smoking does not increase the risk of cSCC. This interpretation is, however, somewhat complicated by several caveats. First was the apparent involvement of a group of participants in a “5-year field trial of daily sunscreen application and beta-carotene supplementation for skin cancer protection” [40]. Secondly, the observations that more females (63%) than males (37%) were lifelong non-smokers (*p* < 0.001), while current smokers were significantly younger than non-smokers and former smokers (*p* < 0.05) [38].

Despite the findings of the latter two negative studies, taken comprehensively, the aforementioned studies are indicative of a link, albeit somewhat tenuous, between smoking and risk of cSCC. In this context, it is plausible that smoking, as opposed to being causative, may potentiate the mutagenic activity of UVR by acting as a cocarcinogen/pro-oxidant while also augmenting the pro-inflammatory/immunosuppressive effects of sun exposure via production of ROS, RNS, and PGE2 [19,41]. Irrespective of potential mechanisms of smoking-related dermatotoxicity, avoidance of the smoking habit, which remains a major public health problem, is clearly non-debatable given its high risk of development of many types of cancer. Nevertheless, stringently controlled biomarker-driven studies are necessary to accurately assess the association of smoking with the pathogenesis of cSCC.

### 3.5. Cutaneous Squamous Cell Carcinoma Associated with Chronic Viral Infection

Infectious agents, including viruses, bacteria, and chronic inflammation, increase the risk of tumorigenesis. The first documented association between squamous cell cancer and chronic infections was shown by Caesar Hawkins in 1835 [42]. This prominent surgeon described seven cases of squamous cell carcinoma arising from chronic osteomyelitis. This observation highlighted the association of chronic inflammation and infection leading to the development of cancer. This relationship was further explored in subsequent research. Peyton Rous first described the association between a viral infection and human cancer in 1911 [43,44,45].

Infection with the predominantly sexually transmitted viruses, human papillomavirus (HPV) and human immunodeficiency virus (HIV), has also been linked to the pathogenesis of cSCC.

#### 3.5.1. Human Papillomavirus

Human papillomavirus is a common skin commensal with a predilection for keratinocytes and, albeit to a lesser extent, for other types of dermal cells. Although an association of cSCC with infection caused by high-risk oncogenic subtypes of HPV belonging to the β-genus had long been suspected, it was not until the advent of nucleic acid-based identification methods that the presence of HPV DNA in biological specimens could be convincingly established. Given the large number of prior inconclusive studies, Wang et al. undertook a meta-analysis focused on the role of HPV in cSCC in which only studies that involved polymerase chain reaction detection of β-HPV subtypes in skin biopsies were included [46]. The objectives of the study, which was published in 2014, were twofold: first was the determination of associations of HPV with cSCC, and, second, a comparison of the HPV DNA loads of tumors from immunosuppressed individuals versus those from immunocompetent individuals [46]. Seventeen studies published during the period 1997–2011 were considered eligible for inclusion in the analysis.

In the first sub-analysis, the authors reported that cSCC lesions were more likely to harbor HPV DNA than normal skin, with the unadjusted and adjusted pooled effect sizes (ES) being 3.43 and 3.12, respectively (both *p* < 0.0001). In the second analysis, tumor biopsies from immunosuppressed participants were associated with significantly increased HPV DNA loads relative to tumors from immunocompetent participants, the pooled ES value being 3.01 (*p* < 0.001) [46].

The authors concluded that HPV skin infection augments the carcinogenic effect of UVR, most prominently in those who are already immunosuppressed [46]. This latter contention is supported by the findings of a pre-clinical study reported by Strickley et al. [47]. These authors observed that immunization of immunocompetent strains of mice via application of the murine commensal papillomavirus type 1 on scarified skin resulted in significant protection against both UVR- and chemical-induced carcinogenesis, an effect that was mimicked by the adoptive transfer of CD8^+^ T cells from immunized to non-immune animals [47]. With respect to the possible clinical relevance of these observations, the authors analyzed human tissue samples (skin lesions and normal skin) for the presence of RNA and DNA derived from 25 different commensal β-HPVs. They observed significant decreases in the viral loads present in lesional skin in comparison with those of normal skin, which they proposed to be indicative of “a strong immune selection against virus-positive malignant cells”. This contention was strengthened by additional observations that CD8^+^ T cells isolated from normal human skin were activated by peptides derived from the E7 oncoprotein of β-HPVs [47]. The findings of this study are consistent with the contention that loss of protective immunity induced by commensal strains of HPV enables high-risk oncogenic strains of the virus to express their pro-tumorigenic potential [47].

These findings reported by Strickley et al. [47] are supported by another very recent murine pre-clinical study reported by Son et al., who observed that immune responses triggered by CD8^+^ dermal T cells against commensal strains of HPV “preserve the homeostasis of highly mutated normal skin” [48]. These authors observed that UVR-induced mutant p53-deficient clones in pre-malignant skin lesions were effectively controlled by HPV-specific CD8^+^ T cells. The authors concluded that the progression and spread of UVR-mutated keratinocytes are restricted by resident commensal skin HPVs via the activation of dermal CD8^+^ T cells, which, in turn, enables maintenance of the stability of the mutated dermis [48].

In this context, an additional, pro-tumorigenic role of HPV has been proposed by Tommasino [49]. This author contends that by acting at an early stage of carcinogenesis, the E6 and E7 oncoproteins of β-HPVs are able to sustain keratinocyte viability and proliferation by attenuating UVR-induced cellular stress, even in the setting of accumulated mutations [49]. Although the underlying mechanisms are unclear, these events, in turn, may also delay progression of keratinocytes to the malignant phenotype [49].

Taken together, these latter three studies indicate that intact CD8^+^ T cell immunity activated by keratinocyte-infected commensal strains of HPV attenuates UVR-induced tumorigenesis in the setting of cSCC by eliminating keratinocytes infected with high-risk, oncogenic strains of the virus [47,48,49]. However, in the setting of severe immunosuppression, this protective mechanism is likely to be significantly compromised, enabling oncogenic strains of HPV to persist and proliferate, augmenting UVR-induced tumorigenesis via several mechanisms. These include, most prominently, but not exclusively, (i) inactivation of p53 by the viral E6 oncoprotein, leading to down-regulation of the *notch* gene (neurogenic locus notch homolog protein 1), a key regulator of tumorigenesis [50], and (ii) E7 oncoprotein-mediated degradation of PTPN14 (protein tyrosine phosphatase non-receptor) via inhibition of LATS1 (tumor suppressor kinase 1) and subsequent activation of YAP1 (yes-associated protein 1) [51,52].

#### 3.5.2. Human Immunodeficiency Virus

It is hardly surprising, given the profound abnormalities of cell-mediated immunity driven by HIV infection, that the risk of cSCC is significantly increased in those living with HIV infection, albeit at much lower incidence rates than those associated with AIDS-defining malignancies [53,54]. This association has been convincingly confirmed in a number of studies from Africa, Europe, and the USA [53,54,55,56,57]. The magnitude of the risk of cSCC associated with each of these studies is as follows:

Standardized incidence risk (SIR) = 3.2, 95% CI 3.2, 95% CI = 2.2–45 (for non-melanomatous skin malignancies, subtypes not indicated) [53].
OR = 2.6, 95% CI = 1.4–4.9 [54].
SIR = 4.64, 95% CI = 3.15–6.03 [55].
Adjusted rate ratio = 2.6, 95% CI = 2.1–3.2 [56].
SIR = 5.4, 95% CI = 3.07–9.52 [57].

In several of those studies, advancing age, high HIV viral loads, and low circulating CD4^+^ T cell counts were associated with higher risk [55,56], while in other studies, prior treatment with highly active antiretroviral therapy (HAART) was evidently not associated with preventive effects [53,57].

In a very recent review article, Reinhart and Leslie expressed concern that the “current guidelines of the American Academy of Dermatology do not address the increased skin cancer risk of people living with HIV beyond Kaposi sarcoma” [58]. They also emphasize the necessity for future quality studies to reliably assess the potential benefit of HAART in reducing the risk of HIV-associated skin cancers [58].

### 3.6. Tattooing

The history of tattooing dates back at least 5000 years, having become popular among British seafarers in the late 18th century, and is now trendy among the youth worldwide [59]. Although an association between tattooing and the development of different types of cutaneous lesions, including skin tumors, keratoacanthoma, and cSCC, has been recognized for several decades, this awareness is largely based on a limited number of small studies and case reports [60]. These are typified by another recent small study reported by Rahbarinejad et al., who described a series of four cases of skin reactions to red-ink tattoos, which included one case of cSCC [61]. Two larger recent studies are, however, noteworthy.

The first of these is a systematic review focused on skin cancers arising within tattoos [62]. Undertaking literature searches from the inception of the selected databases up until 23 February 2023, the authors identified 105 eligible studies comprising 160 cases of cutaneous tumors arising within tattoos. Twenty of these were cases of cSCC, 55% of which developed within the tattoo red-ink pigment [62].

The second study, a Swedish population-based case–control study undertaken by Leljedahl et al. focused on tattooing as a risk factor for both cutaneous melanoma (n = 2880 cases) and cSCC (n = 2859) in these two cohorts diagnosed between 2014 and 2017 in a country where 20% of the population is tattooed [63]. The authors’ analyses revealed that tattooing was associated with an increased risk for cutaneous melanoma [incidence rate ratio (IRR) = 1.23, 95% CI, 1.06–1.44] compared with non-tattooed individuals. This was mainly driven by superficial spreading melanoma (IRR = 1.37, 95% CI, 1.11–1.69). However, no association of tattooing with an increased risk of cSCC was evident (IRR = 0.86, 95% CI, 0.73–1.02) [63]. Potential caveats to explain the observed lack of association between tattooing and risk of cSCC in this study may relate, firstly, to low-level exposure to UVR in this geographic region, given that interactive effects between tattooing and sun exposure may be relevant. Secondly, there is a possible lack of awareness of the potential influence of the apparent significance of red-ink-based tattoos.

The risk factors mentioned in this section of the review, both proven and suspected, are summarized in Table 1. These risk factors should, however, be viewed collectively, rather than in isolation.

## 4. Differential Gene Expression, Driver Mutation Profiling, and Role of Neoantigens in Cutaneous Squamous Cell Carcinoma

### 4.1. The Genomic Landscape

UVR-mediated DNA damage and repair occur variously across the genome; for example, UVR-induced pyrimidine dimers are repaired more effectively in genes that are actively transcribed relative to inactive genomic regions or the entire genome [64,65].

Several studies have attempted to identify genes that are differentially expressed in established cSCC relative to those expressed in normal skin and pre-malignant lesions. Wei et al. applied microarray analysis to probe this issue [66]. Following analyses of genes (n = 833) from healthy tissue and lesional samples derived from two different datasets, only three genes, namely, *KRT16* (keratin 16), *PI3* (peptidase inhibitor 3), and *EGR3* (early growth response 3), were largely found to be differentially expressed [66]. However, only *EGR3* had the same expression pattern in both datasets and was found to be most prominently expressed in cSCC. However, as opposed to being a key driver gene, the authors proposed that upregulated expression of *EGR3* represented a biomarker with both diagnostic and prognostic potential [66].

In a later study, Zou et al. used a transcriptome sequencing and bioinformatics platform to explore the differential expression of genes (n = 46,930) in tissue samples from pre-cancerous actinic keratosis and cSCC, as well as from sun-exposed and non-sun-exposed skin taken from 24 participants [67]. Five genes were identified as being significantly upregulated in cSCC. These were *HEPHL1* (hephaestin-like 1), *FBN2* (fibrillin 2), *SULF1* (heparan sulfate endosulfatase), *SULF2* (extracellular sulfatase), and *TCN1* (transcobalamin 1) [67]. Once again, however, rather than assigning key driver status to these genes, the authors acknowledged their potential as probable biomarkers and therapeutic targets [67].

As is evident from the prior two studies and highlighted by Chang et al., distinguishing driver mutations from passenger mutations represents a daunting challenge given the extremely high mutation burden in cSCC, particularly in the setting of solar UVR-induced DNA damage [68]. This issue is further complicated by the ongoing accumulation of solar UVR-induced somatic mutations from an early age, together with attenuation of the efficacy of DNA-repair mechanisms with older age [69].

As a strategy to overcome this challenge, Chang et al. performed a “meta-analysis” of publicly available sequencing data covering 105 cSCC tumors (88 in the final analysis) from ten different studies [68]. Tumor subtypes included those from patients with xeroderma pigmentosum, spontaneous tumors from otherwise healthy individuals, those from immunosuppressed patients receiving azathioprine or alternative immunosuppressants, and patients with recessive dystrophic epidermolysis bullosa. Studies identified via literature searches were those that included “whole exome or whole-genome sequencing of a cSCC in which raw data were made publicly available” [68]. The identification of priority driver genes was based on “state-of-the-art cancer gene discovery algorithms”, of which 30 genes were nominated for detailed analysis. The analysis revealed, not unexpectedly, that mutations in the Notch and p53 pathways were common, while, albeit to a lesser extent, mutations in the following pathways were also evident: the Hippo signaling pathway, the Ras/MAPK/PI3K (RasGTPase/mitogen-activated protein kinase/phosphoinositide 3-kinase) pathways, and genes involved in checkpoint control and chromatin remodeling [68]. Novel, frequently mutated genes included *EP300* (encodes a histone acetyltransferase), *PBRM1* (polybromo1, tumor suppressor), *USP28* (ubiquitin-specific peptidase 28), and *CHUK* (conserved helix–loop–helix ubiquitous kinase) [68]. The authors concluded that their study “provides the most detailed catalog of driver genes in cutaneous squamous cell carcinoma to date,” enabling mechanistic insights into the fundamental biology of cSCC, as well as potential therapeutic targets [68]. Nevertheless, further detailed analysis is required not only to establish the hierarchy of the identified driver genes but also to uncover additional genes and to probe the mechanisms and types of driver genes that trigger advanced disease. Furthermore, validation of the clinical utility of the detection of upregulated expression of the aforementioned genes remains to be confirmed in large prospective studies.

### 4.2. Tumor Neoantigens (Tumor-Specific Antigens)

Cutaneous SCC has an extremely high, dynamic mutational load, with a median of almost 50 mutations per megabase pair [70,71]. This ongoing mutational burden, in turn, leads to the frequent formation of neoantigens, which arise from mutated somatic genes and are entirely tumor-specific [72,73]. They are displayed on the tumor cell surface in the context of major histocompatibility complex (MHC) molecules, where they interact with complementary T cell receptors (TCRs) present on anti-tumor infiltrating T lymphocytes (TILs).

The dynamic mutational burden characteristic of cSCC, as well as actinic keratosis, is accompanied by the progressive expansion of a repertoire of these neoepitopes, which have the potential to activate protective anti-tumor cytotoxic CD8^+^ T cell-mediated immune mechanisms, as mentioned above [74,75]. Clearly, immunocompetent persons are the primary beneficiaries of these protective immune mechanisms, a benefit that is negated in the setting of defective cell-mediated immunity, possibly augmented by immune exhaustion driven by over-simulation due to the substantial repertoire of reactive neoepitopes. This latter contention is supported by the findings of Pham et al. [76]. These authors reported that UVR-induced mutagenesis results in the formation of hydrophobic neoantigens with increased immunogenicity due to augmentation of interaction with MHC class I [76]. The authors investigated relationships between the protective efficacy of neoantigen hydrophobicity and low/intermediate and high tumor mutational burdens (TMB) in people with melanoma (n = 151) treated with programmed cell death protein-1 (PD-1)/PD-L1-targeted immunotherapy. They observed that only patients in the low/intermediate TMB group demonstrated significant clinical improvement [76]. This counterintuitive finding may have been attributable to an excessive/exhaustive immune reactivity in the high-TMB group [76].

In summary, the types of mutagenic agents and exposure levels, as well as the spectrum, physical properties, and immunogenicities of the resultant neoantigens, together with the immune competence of the cSCC-afflicted host, all appear to be determinants of tumor progression.

## 5. Mechanisms of Immune Evasion in Cutaneous Squamous Cell Carcinoma

Tumors are remarkably adept at subverting mechanisms of anti-tumor immunity, thereby perpetuating tumor cell survival and enabling progression and spread. Pro-tumorigenic mechanisms of immune evasion have been intensively investigated in the context of many different types of malignancy. However, mostly because of the highly localized, slow-growing, minimally invasive nature of cSCC, comparatively few studies have explored mechanisms of immune evasion in the setting of this dermatological malignancy [77,78]. In distinction to the latter studies, the current study represents an update of studies published in the last four years, which are largely focused not only on immune profiling of the tumor microenvironment (TME) but also on the identification of potential mechanisms of systemic immunosuppression.

### 5.1. Mechanisms of Immune Evasion Operative in the Tumor Microenvironment

To explore alterations in cytokine/chemokine (n = 24) profiles at various stages in the progression of cSCC, Tuong et al. compared the levels of these inflammatory mediators in homogenized biopsies (n = 67, 58 histologically well-differentiated) from participants with actinic keratosis and cSCC relative to those of healthy skin [79]. The levels of the 24 test cytokines/chemokines were mostly comparable with respect to the actinic keratosis and cSCC sub-groups, but significantly increased relative to those of healthy skin [79]. However, in the case of cSCC, the authors established the involvement of upregulated CCR5 ligands, as well as that of several other chemokines, including CXCL9, CXCL10, and CXCL11, in maintaining a state of immunosuppression during well-defined stages of cSCC progression [79]. These pro-inflammatory chemokines are likely to underpin the influx of various types of precursor immune suppressor cells into the TME, where they undergo maturation into suppressive invariant γδ T cells, Tregs, T helper 2 (Th2) lymphocytes, M2-type macrophages, cancer-associated fibroblasts (CAFs), and type 2 neutrophils [80,81].

### 5.2. Transforming Growth Factor-β1

Transforming growth factor-β1 plays contrasting dual roles in the development of cSCC. Initially, this cytokine inhibits the growth of keratinocytes in healthy skin [82]. However, this key regulatory function of TGF-β1 is compromised following UVR-induced mutations in essential genes of the signal transducing receptors of the TGF-β superfamily (SMAD), specifically TGF-β receptor 1 (*TGFβRI*), *TGFβRII*, *SMAD2*, and *SMAD4* [82]. These events not only result in loss of TGF-β1 regulation of cell growth and oncogenesis, but also in significant over-expression of TGF-β1 in the TME, which, via various mechanisms of immune evasion, contributes significantly to tumor growth and progression [82].

The cellular origins of TGF-β1 in the TME include the tumor per se, as well as infiltrating immune and inflammatory cells, including neutrophils, macrophages/monocytes, and Tregs in particular, with the cytokine acting via both autocrine and paracrine signaling. In addition to pro-fibrotic activity and the promotion of epithelium-to-mesenchymal transition (EMT), TGF-β1 also promotes the differentiation of Tregs and type 2 phenotype polarization of macrophages and neutrophils [83,84,85,86,87]. These cells, in turn, orchestrate production of additional mediators of immunosuppression, most prominently, adenosine, IL-10, and PGE2 [88,89].

The key roles of TGF-β1 and PGE2 in sustaining an immunosuppressive milieu in the TME are highlighted by an, albeit small, phase I “single-center, open-label, dose-escalation study” undertaken in patients with cSCC in situ. The authors evaluated the safety and efficacy of the intra-lesional administration of a TGF-β1/cyclooxygenase-2 (COX-2) small interfering RNA (siRNA) combination known as STP705 [90,91]. The two types of siRNA form a complex with a biodegradable copolymer (HKP) enriched with histidine and lysine residues, packaged in nanoparticles, which facilitate tumor uptake [91]. With respect to the cSCC clinical trial, the authors recruited 25 patients, divided into 5 cohorts of equal numbers, each injected intra-lesionally with an escalating dose of STP705 up to a maximum of 120 micrograms (μg) weekly for 6 weeks [90]. The primary endpoint was the proportion of patients who manifested complete pathological clearance. Collectively (all 5 cohorts), 76% (n = 19) of patients achieved clearance in the 30 and 60 μg treatment cohorts, respectively [90]. Seven adverse events, which were considered to be neither “severe or serious”, were reported in five patients. The authors contend that STP705-based intra-lesional combination immuno-/anti-inflammatory therapy is “non-invasive, safe and efficacious in treating cutaneous in situ squamous cell carcinoma” [90]. In addition, their findings also underscore the prominence of TGF-β1 and PGE2 as being significant mediators of immune evasion in the TME of cSCC.

These findings have been partially replicated in a very recent pre-clinical study in which the authors used an implanted, syngeneic, orthotopic model of hepatocellular carcinoma in mice [92]. In this study, the TGF-β1/COX-2 siRNA combination, prepared as a liver-targeted polypeptide nanoparticle formulation, was injected at a dose of 2 milligrams/kilogram (mg/kg) into the tail vein of tumor-bearing mice (six doses over a 3-week period). Complete inhibition of further tumor growth was observed following administration of the TGF-β1/COX-2 siRNA combination. This was associated with augmentation of anti-tumor immunity characterized via increased infiltration of CD4^+^ and CD8^+^ T cells [92].

Ongoing clinical studies are described below.

### 5.3. Adenosine

The production of the potent immunosuppressive nucleoside adenosine by infiltrating Tregs, of which there are several subtypes, represents another potential mechanism of immune evasion operative in the cSCC TME [80,93,94]. Although largely unexplored in the setting of cSCC, a brief overview of the immunosuppressive activities of adenosine is nevertheless relevant, given its potential importance as a target for anti-tumor immunotherapy.

In the case of Tregs, high-level expression of the ectoenzymes ectonucleoside triphosphate diphospho-hydrolase-1 and ecto-5′-nucelotidase, known as CD39 and CD73, respectively, enables Tregs to metabolize extracellular adenosine-5′-triphosphate (ATP) to adenosine. Unlike effector T cells, which express high-level activity of the adenosine-neutralizing enzyme, adenosine deaminase (ADA) in association with its cell membrane receptor, CD26 (dipeptidyl peptidase 1), Tregs do not retain ADA due to low-level/absent expression of CD26 [95], resulting in the substantial synthesis and extracellular release of adenosine.

Acting via adenylyl cyclase subtype A2A receptors, adenosine has been reported to promote the polarization of M1 and N1 phenotype macrophages and neutrophils to the suppressive M2 and N2 phenotypes, respectively [96,97]. In addition, acting via subtype A2B receptors, adenosine has been reported to drive (i) a pro-tumorigenic Th1→Th2 switch [98] and (ii) angiogenesis via interaction with vascular endothelial cells, resulting in cell proliferation and the release of vascular endothelial growth factor, promoting metastasis and disease progression [99].

Although a paucity of publications on the possible involvement of adenosine as a mediator of immune evasion in cSCC currently exists, two reports in patients with HNSCC are noteworthy. In the first of these, Chandrakiran et al. reported that elevated serum levels of adenosine-neutralizing ADA are significantly correlated with disease severity in head and neck cancer, declining in response to successful treatment, and are superior to tumor HPVC status as a predictive biomarker [100]. Secondly, Huo et al. in their recent review, described the types and consequences of adenosine-mediated modifications to RNA in relation to the development, progression, and treatment of HNSCC [101].

In the case of the involvement of adenosine in the immunopathogenesis of cSCC, a recent study reported by Whitley et al. is noteworthy [102]. The authors described substantial, selective, peritumoral infiltration of immunosuppressive, CD39-expressing FoxP3^−^ and FoxP3^+^ T cells into human cSCCs, which was associated with high levels of extracellular adenosine, particularly in tumors that had metastasized [102]. The FoxP3^−^ T cell population also encompassed a subset of immunosuppressive CD39/PD-1 co-expressing cells, representing a potentially significant source of attenuation of anti-tumor immune mechanisms. These studies on human cSCCs were complemented by the findings of an experimental murine model of UVR-induced carcinogenesis, which revealed that increased T cell expression of CD39 was associated with the induction of synthesis of IL-27. Additionally, in vitro studies demonstrated that exposure of cultured keratinocytes and a cell line (A431) to UVR resulted in down-regulation of NAP1L2 (nucleosome assembly protein 1L2), which is involved in the repair of UVR-induced DNA damage [102]. Although further studies are warranted, these observations underscore both the immunosuppressive and mutagenic potential of adenosine in the pathogenesis of cSCC.

### 5.4. Attenuation of Expression of Highly Immunogenic Neoepitopes

A recent study undertaken by Borden et al. compared the immunogenicity of neoantigens sampled from actinic keratosis and cSCC lesions (seven of each, taken from nine patients) [74]. The study necessitated measurement of the relative binding strengths to MHC class I and TCRs of neoantigens present in the lesional samples. These were identified in datasets based on whole-genome and RNA-sequencing analyses of the lesional samples, together with application of a fitness cost model [74]. The authors observed that although the numbers of mutations and neoantigens were comparable between the sampled actinic keratosis and cSCC lesions, the predicted immunogenicity of the highest expressed-adjusted fitness cost was found to be lower for cSCC [74]. These observations are indicative of tumor-mediated down-regulation of the most immunogenic neoantigens in cSCC as an additional mechanism of immune evasion [74].

### 5.5. Systemic Immunosuppression

Because of the highly localized nature of cSCC, relatively little research appears to have been undertaken with respect to the issue of identifying systemic mechanisms of immunosuppression. This contrasts with BCC, which is associated with significantly increased systemic levels of immunosuppressive TGF-β1, as well as those of the soluble co-inhibitory immune checkpoints, cytotoxic T-lymphocyte antigen-4 (CTLA-4), lymphocyte activation gene 3 (LAG-3), PD-1, PD-L1, and T cell immunoglobulin and mucin domain-containing protein-3 (TIM-3) [103]. One study, as elaborated on below, has, however, reported that an increased frequency of the circulating chemokine receptor 4 (CCR4^hi^)-expressing Treg subtype is associated with a substantially increased risk of the development of incident cSCC [104].

**Figure 1:**
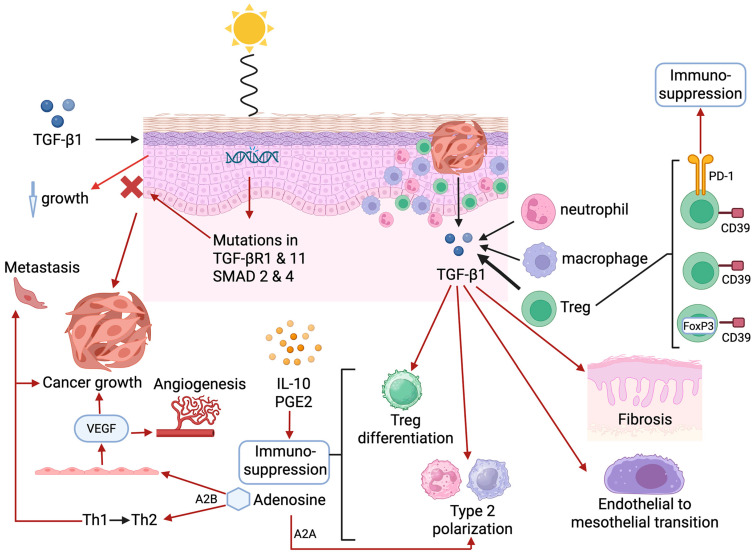
Molecular changes leading to cutaneous squamous cell carcinoma development. TGF-β1 inhibits the growth of keratinocytes in healthy skin, but UVR-induced mutations in essential genes (*TGFβRI*, *TGFβRII*, *SMAD2,* and *SMAD4*) result in loss of TGF-β1 regulation of cell growth and oncogenesis. TGF-β1 is also over-expressed in the tumor microenvironment, originating from the tumor and infiltrating immune and inflammatory cells, including neutrophils, macrophages/monocytes, and Tregs in particular. TGF-β1 has pro-fibrotic activity and promotes epithelium-to-mesenchymal transition, as well as differentiation of Tregs and type 2 phenotype polarization of macrophages and neutrophils. These cells produce additional mediators of immunosuppression, especially adenosine, IL-10, and PGE2. Tregs synthesize and release adenosine. Acting via adenylyl cyclase subtype A2A receptors, adenosine promotes the polarization of M1 and N1 phenotype macrophages and neutrophils to the suppressive M2 and N2 phenotypes, respectively. Acting via subtype A2B receptors, adenosine drives a pro-tumorigenic Th1→Th2 switch and angiogenesis via interaction with vascular endothelial cells, resulting in cell proliferation and release of VEGF, promoting metastasis and disease progression. Infiltrating Tregs consist of various subtypes, the most notable being CD39-expressing FoxP3^+^ Tregs and FoxP3^−^ Tregs expressing CD39 with or without PD-1, believed to represent a potentially significant source of attenuation of anti-tumor immune mechanisms.

These various mechanisms of TME-associated and systemic immunosuppression operative in cSCC are summarized in Table 2 and pictorially in Figure 1.

## 6. Systemic Chemotherapy, Targeted Therapy, and Immunotherapy

Chemotherapy, targeted therapy drugs (EGFR inhibitors), and immune checkpoint inhibitors (ICIs) are the main treatment options available for cSCC that have spread to lymph nodes or distant organs [105]. These types of treatment might be administered as a single modality or in combination.

### 6.1. Systemic Chemotherapy and Targeted Treatments

Cisplatin-based chemotherapy was historically associated with short-lived responses with significant toxicities. Cisplatin-based chemotherapy is associated with a poor response to treatment and a high toxicity rate, with a 4-year survival rate of 6% [106]. However, due to the overall efficacy and safety of immunotherapy, this new treatment modality makes it the preferred choice in cases where curative surgery or RT are not viable.

Before the development and availability of ICIs, advanced cSCC was primarily treated with targeted treatments using anti-EGFR monoclonal antibodies (mAbs) [107]. Cetuximab is an anti-EGFR mAb used to treat cSCC. A retrospective, multicenter study investigated cetuximab, demonstrating moderate efficacy in patients with metastatic or locally advanced cSCC [108,109]. The median progression-free survival was 9.7 months, and the overall survival was 17.5 months. However, with the introduction of immunotherapy, the usage of EGFR-targeted therapies has become less prominent. Nevertheless, cetuximab is often utilized as a secondary-line option following primary or acquired resistance to ICIs [72,110].

### 6.2. Immunotherapy

The history of immunotherapy goes back to the late end of the XIX century, with William Coley’s work on using bacteria to treat cancer and Paul Ehrlich’s immune surveillance theory. Coley observed that cancer patients experienced remission after infections. He developed the “Coley’s toxin” by injecting heat-killed bacteria into cancer patients. He reported results of 140 advanced sarcoma patients in 1898 [111]. Paul Ehrlich was a Nobel Prize-winning German physician and scientist who hypothesized that the immune system actively monitors and eradicates malignant cells [112].

James P. Allison and Tasuku Honjo were awarded the 2018 Nobel Prize in Physiology or Medicine for their groundbreaking work on cancer immunotherapy. Their investigations primarily centered on the inhibition of negative immune regulation pathways. Developing mAbs that block inhibitory immune pathways allows the immune system to attack cancer cells effectively [113]. This work led to the development of anti-PD-1 and other immune checkpoint inhibitors in the treatment of many malignancies, including cSCC.

For advanced SCCs that cannot be cured with surgery or RT, ICI treatment with cemiplimab (Libtayo^®^), pembrolizumab (Keytruda^®^), or cosibelimab (Unloxcyt^®^) is currently the preferred option.

Cemiplimab is a high-affinity PD-1-targeted mAb. This was the first agent approved by the United States Food and Drug Administration (FDA) and the European Medicines Agency (EMA) for treating locally advanced and metastatic cSCC, administered intravenously every three weeks at 350 mg per infusion [114,115]. The registration of cemiplimab was based on the EMPOWER-CSCC-1 multiple-cohort phase II study in patients with cSCC. Two cohorts received intravenous cemiplimab at 3 mg per kg every 2 weeks, while another two cohorts received intravenous cemiplimab at 350 mg every 3 weeks. At 42.5 months, the objective response rates for groups of 193 patients receiving 3 mg per kg were 47%, with a median progression-free survival (PFS) of 26.0 months. In the second group (n = 165 patients) treated with intravenous cemiplimab 350 mg every 3 weeks, the objective response rate was 44%, while a median PFS was not reached. Grade 3 or 4 immune-related adverse events were documented in approximately one-third of the patients. The EMPOWER-CSCC-1 is the most extensive prospective study showing long-term efficacy and safety for an ICI in patients with advanced cSCC [115].

Additionally, a retrospective, observational, multicenter study from Italy analyzed the medical records of advanced cSCC patients treated with cemiplimab in 17 referral centers. This study demonstrated an overall response rate of 58%, and the disease control rate was 72% [116].

Pembrolizumab was associated with a robust, durable antitumor activity in locally advanced and relapsed cSCC. The phase II KEYNOTE-629 study, in which 200 mg was administered every 3 weeks for up to 35 cycles, established pembrolizumab as a treatment option for cSCC in this setting. The study enrolled 159 patients, consisting of a cohort of 54 patients with locally advanced cSCC and a relapsed metastatic cohort of 105 patients. In the locally advanced cohort, the objective response rate was 50.0% [95% CI, 36.1% to 63.9%]. These responses included 16.7% of patients with a complete response (CR) and 33.3% with a partial response (PR). The OR in the relapse or metastatic cohort was 35.2% (95% CI, 26.2% to 45.2%), including 10.5% of patients with a CR and 24.8% with a PR. The toxicity profile with pembrolizumab was generally consistent with its established safety profile [117,118].

Amatore et al. [119] conducted a phase II single-arm neoadjuvant clinical trial of pembrolizumab in untreated resectable locally advanced cSCC. Patients with AJCC/UICC ≥T3 or high-risk T2 (tumor diameter ≥ 2 cm, poorly differentiated histology, perineural invasion ≥ 0.1 mm, or tumor invasion beyond fat) and/or N+ disease were eligible [120]. Patients received two cycles of pembrolizumab (200 mg every 3 weeks) prior to definitive surgery, followed by 15 cycles of pembrolizumab post-surgery (NCT04808999). The primary endpoint was a pathological complete response (pCR). A selected panel of multiplex immunofluorescence imaging biomarkers (PredxBio Inc. computational pipeline) was also used to evaluate the TME. A total of 30 patients were enrolled, and 26 patients were evaluable. The median age was 79, with mainly male patients (77%). Of the 26 response-evaluable patients, pCR was achieved in 15 patients (57%); postoperative RT was de-escalated in 14 of the 15 patients who attained a pCR. Computational analysis identifies key spatial interactions between PD-L1+/CD68+ cells implicated in the response to pembrolizumab. The authors concluded that neoadjuvant pembrolizumab is an effective treatment in resectable locally advanced cSCC with a high pCR rate (57%). These encouraging results need to be confirmed in a prospective randomized, adequately powered phase III study [119].

Cosibelimab is a third anti-PD-1 antibody, recently registered by the FDA for the treatment of metastatic cSCC. The registration was based on a phase II study. Participants with metastatic cSCC received cosibelimab 800 mg intravenously every 2 weeks. The primary endpoint was the objective response rate using Response Evaluation Criteria in Solid Tumors, Version 1.1 [121]. In this study, objective responses were observed in 37 of 78 participants (47.4% with a 95% CI of 36.0% to 59.1%). These authors demonstrated that cosibelimab attained clinically meaningful responses and had a manageable safety profile [114].

Preventive strategies to ameliorate the development of cSCC in immunosuppressed patients include (i) the use of protective clothing and sunscreens; (ii) more frequent follow-ups by experienced dermatologists; (iii) patient education and self-examination; (iv) the judicious use of immunosuppressive agents; and (v) prophylaxis with retinoids including acitretin [122].

Future studies should investigate the role of anti-PD-1 antibodies in combination with chemotherapy or targeted therapy. Additionally, as described above, the intra-lesional administration of STP705 in combination with anti-PD-1 mAbs could potentially improve outcomes in patients with locally advanced cSCC [90]. Another potential clinical strategy to ameliorate adenosine-mediated immunosuppression, albeit unexplored to our knowledge, involves the intra-lesional administration of stabilized adenosine deaminase. Refining the role of biomarkers for better patient selection is also of paramount importance. In addition to the biomarkers mentioned in the following section, our group has demonstrated the potential immunosuppressive roles of plasma TGF-β1 and soluble, systemic co-inhibitory immune checkpoint molecules (ICMs) in patients with BCC [103]. Future studies should address the potential utility of these same biomarkers in cSCC.

## 7. Potential TME-Associated and Systemic Biomarkers in Cutaneous Squamous Cell Carcinoma

The incidence of cSCC, together with the risk of metastasis and recurrence, underscores the necessity to find biomarkers that can assist clinicians diagnostically, prognostically, and with the selection of patients, as well as for monitoring of treatment. However, although candidate biomarkers of TME origin have been identified, the most promising of these remain to be convincingly validated. Disappointingly, a similar situation exists with respect to systemic biomarkers, which are less invasive, cost-effective, and clearly require further research in the setting of cSCC.

Most cSCC candidate biomarkers of lesional origin have centered around histological and molecular signals, both genomic and transcriptomic, as discussed above and recently reviewed by Montano et al. [123]. In addition to these, the authors list a number of protein markers of lesional origin in both cSCC and BCC that have been identified as having possible prognostic, diagnostic, and predictive utility, which again require confirmation in the clinical setting [123,124,125,126,127,128,129,130,131,132].

Examples of potential, albeit unvalidated, biomarkers of TME and systemic origin are listed in Table 3 below. This is followed by a more detailed discussion of potentially useful systemic biomarkers.

### Novel Potential Systemic Biomarkers

Blood-based liquid biopsy techniques are attractive tools for assessing tumor heterogeneity, monitoring tumor development during immunotherapy, and determining residual tumor load. They have the added benefit of being minimally invasive and can be measured sequentially [4]. Important examples of biomarkers of this category include peripheral Tregs, circulating tumor cells (CTCs), circulating tumor DNA (ctDNA)—also known as cell-free DNA (cfDNA)—and extracellular vesicles (EVs), each of which is briefly discussed below.

In this context, UVR exposure has been found to have a positive correlation with circulating Tregs. As mentioned above, Rollison et al. [104] conducted a study examining the relationship of circulating Tregs with cSCC risk, particularly in high-UVR-exposure settings. In a prospective cohort study, involving 327 immunocompetent participants, routine skin cancer screenings, blood samples, and estimates of UVR exposure were taken at the outset of the study, and individuals were monitored for incident cSCC for up to 4 years. A greater proportion of CCR4^hi^ peripheral Tregs predicted incident cSCC occurrence, especially among individuals highly exposed to UVR. The authors proposed that further investigation into the underlying biology of Tregs in UVR-related skin damage could potentially uncover new avenues for screening, prevention, and treatment strategies.

Circulating tumor cells are viable, intact cancer cells that originate from primary tumors or metastatic sites and circulate in the bloodstream at low levels. These cells can be quantified and analyzed for biomarker expression. While CTCs have demonstrated potential in melanoma and HNSCC, the clinical utility of their measurement in cSCC remains to be confirmed [4].

Circulating tumor DNA is the fraction of cell-free DNA in blood that originates from tumor cells. It is primarily studied in plasma, though it can be found in other bodily fluids such as saliva. The ability to detect selective tumor mutations makes ctDNA a promising tool for patient management. Unlike HNSCC, where ctDNA has shown utility, its application in cSCC remains to be established [4]. In this context, Fan et al. presented their initial data in relation to the measurement of ctDNA in patients with cSCC at the 2024 American Society of Clinical Oncology meeting. They assessed 21 samples submitted for Natera ctDNA analysis and concluded that ctDNA is a reliable marker for patients with a high tumor volume [133].

Extracellular vesicles are small, membrane-enclosed structures released by numerous cell types, including tumor cells, and can be identified in various bodily fluids, including blood [135]. EVs are promising candidates for cancer liquid biopsy due to their accessibility, stability, and resilience compared to CTCs and cfDNA. These vesicles comprise proteins, RNA, DNA, and lipids, which are selectively incorporated from their cells of origin and play roles in cancer progression, immune modulation, metastasis, and drug resistance [135]. Studies have demonstrated their potential as biomarkers for hepatocellular carcinoma; pediatric solid tumors; and cancers of the pancreas, ovary, and bladder [137,138,139]. While limited research exists in cSCC, a systematic review by Lee et al. [134] encompassing seven pre-clinical and clinical studies highlighted the biomarker potential of post-translationally modified desmoglein 2 (Dsg2). This protein is a component of the desmosomal epithelial cell-to-cell adhesion structure and may represent a useful biomarker of cSCC in the future.

In addition, EVs contain many microRNAs (miRNAs), which are small, endogenous non-coding RNAs that can influence cellular processes of apoptosis, invasion, proliferation, or migration by means of post-transcriptional regulation of gene expression [4]. The potential roles of miRNAs in the management of cSCC have recently been reviewed by Natarelli et al. [136]. High-throughput profiling of non-coding RNAs and/or proteins shows considerable promise in identifying potential biomarker signatures associated with response to treatment in cSCC.

Although these biomarkers described above show considerable promise, their sensitivities, specificities, and utility remain to be demonstrated in large, adequately powered clinical trials. Notably, novel biomarkers will probably be used in conjunction with established diagnostic standards rather than replacing them. [4]

## 8. Conclusions

It is well-recognized that prolonged and even episodic exposure to UVR, augmented by factors such as genetic predisposition, harmful aspects of lifestyle, and underlying disease-associated immune dysfunction, underpin the mutational burden and immunosuppression that drive the development of cSCC. While the search continues for accurate identification of a key driver mutation, or combinations thereof, the role of tumor-orchestrated immune evasion in promoting disease persistence and progression is becoming increasingly evident. Understanding these mechanisms of immune evasion, in turn, provides a significant cornerstone for the identification of both novel therapeutic targets and biomarkers of disease severity and response to treatment. In this context, the prominent TME-derived mediators of immunosuppression, TGF-β1, PGE2, and possibly adenosine, as well as tumor-infiltrating and systemic Tregs, represent key pro-tumorigenic drivers of immune evasion. Given the notable, albeit limited, success of PD-1-targeted immunotherapy in the treatment of established/advanced cSCC, future research in this context is likely to involve assessment of the immunotherapeutic efficacy of the dual administration of inhibitory ICM-targeted mAbs with novel intra-lesional agents such as STP705. Future strategies focused on the identification of predictive/prognostic biomarkers include high-throughput proteomic and non-coding RNA profiling, as well as multiplex determination of the systemic levels of soluble co-inhibitory ICMs.

## Figures and Tables

**Table 1:** Proven and suspected risk factors for cutaneous squamous cell carcinoma.

Risk Factor	Mechanism
Potentially avoidable occupational and recreational excessive exposure to ultraviolet radiation	Most prominent risk factor due to potent pro-mutagenic and pro-inflammatory/immunosuppressive activities of UVR [1,2,3,4,5,19,21,22,23,24].
Radiation therapy	A cornerstone treatment of various types of malignancy, including cSCC, in which the potential risk is clearly outweighed by clinical benefit [25,26,28].
Iatrogenic immunosuppression	Promotes considerable augmentation of the intrinsic immunosuppressive activity of solid malignancies [4,29,30].
Smoking	Appears to augment the pro-mutagenic, immunosuppressive effects of UVR, but conflicting reports exist [33,34,35,38].
Human papillomavirus infection	A double-edged sword in which cell-mediated immune responses to commensal skin HPV augment the survival of UVR-mutated keratinocytes. Progression of cSCC is, however, augmented by oncogenic strains of the virus in the setting of defective cell-mediated immunity [46,47,48,50,51,52].
Human immunodeficiency virus	Progression of cSCC driven by defective cell-mediated immunity [53,54,55,56,57,58].
Tattooing	Possible augmentative pro-tumorigenic activity, particularly in the setting of red-ink tattoos acting as a potential dermatotoxin [60,61,62,63].

Abbreviations: cSCC, cutaneous squamous cell carcinoma; HPV, human papillomavirus; UVR, ultraviolet radiation. Relevant references are shown in parentheses.

**Table 2:** Prominent mechanisms/mediators of immune evasion operative in the tumor microenvironment and circulation of cutaneous squamous cell carcinoma.

Mediators/Site	Mechanisms of Immune Evasion
TME	Influx of various types of immunosuppressive cells expressing ligands interactive with CCR5 (macrophages/monocytes, neutrophils, Tregs, Th2 cells, and invariant Ɣδ T cells) [79,80,81].
Transforming growth factor-β1	- Pro-fibrotic - Promotes epithelium-to-mesenchymal transition - Promotes differentiation of Tregs - Promotes type 2 phenotype polarization of type 1 phenotype macrophages and neutrophils, resulting in production of immunosuppressive IL-10 and PGE2 [82,83,84,85,86,87,88,89,90].
Adenosine	Potent broad-spectrum immunosuppressive and pro-mutagenic nucleoside produced by CD39-expressing infiltrating T cells [95,96,97,98,99,102].
Down-regulation of expression of highly immunogenic neoepitopes	Results in attenuation of tumor recognition by CD8^+^ cytotoxic T cells [76].
Circulation	Increased numbers of pro-tumorigenic CCR4^hi^ Tregs [104].

Abbreviations: CCR, chemokine receptor; IL, interleukin; PG, prostaglandin; Th2, type 2 helper lymphocyte; TME, tumor microenvironment; Treg, regulatory T cell. Relevant references are shown in parentheses.

**Table 3:** Summary of biomarkers associated with the tumor microenvironment (TME) and potential systemic biomarkers.

Biomarker	Significance in cSCC
**TME-associated:**	
Immune checkpoint molecules	
PD-1	Expressed in locally advanced and metastatic cancer [124,131].
LAG-3	Expressed by a subset of TILs, specifically CD8^+^ T cells [124,125].
Matrix metalloproteinases	
MMP-2	Upregulated expression in cSCC [126].
MMP-7	Promotes the growth of cSCC by shedding HB-EGF, leading to the activation of EGFR [127].
MMP-9	Upregulated expression in cSCC [126].
MMP-13	Potential early detection of invasiveness and monitoring progression of cSCC [128].
Complement components	
C1r and C1s detected by increased levels of mRNA	Elevated levels in cSCC cells linked to inhibition of ERK1/2 and PI3K signaling pathways, promoting apoptosis of cSCC cells, and suppressing vascularization and growth of cSCC xenografts in vivo [129].
TILs	
Tregs	Larger Treg population in pre-metastatic lesions and a reduction in Treg number in invasive cSCC [130].
CD8^+^ T cells	Lower numbers of CD8^+^ T cells due to TGF-β1 inducing the expression of T cell exhaustion markers such as PD-1, TIM-3, and CTLA-4 [132].
**Systemic**	
Peripheral Tregs	Greater proportion of CCR4^hi^ peripheral Tregs predictive of incident cSCC, particularly in individuals highly exposed to UV radiation [104].
CTC	Can be quantified and analyzed for immune-marker expression [4].
ctDNA/cfDNA	Selective tumor mutations can be detected [133].
EVs	Comprise proteins, RNA, DNA, and lipids that are selectively incorporated from their cells of origin [134,135].Post-translational modification of desmoglein 2, a component of the desmosomal cell-to-cell adhesion structure may assist with characterization and treatment of cSCC [134].
miRNAs	Influence cellular processes of apoptosis, invasion, proliferation, or migration by means of post-transcriptional regulation of gene expression [4,136].

Abbreviations: cfDNA, cell-free DNA; CTC, circulating tumor cells; ctDNA; circulating tumor cell DNA; CTLA-4, cytotoxic T-lymphocyte antigen 4; EGFR, epidermal growth factor receptor; ERK1/2, extracellular signal-related kinase 1/2; EVs, extracellular vesicles; HB-EGF, heparin-binding epidermal growth factor; LAG-3, lymphocyte activation gene 3; MMP, metalloproteinase; mRNA, messenger ribonucleic acid; miRNA, micro RNA; PD-1, programmed cell death protein-1; PI3K, phosphoinositide 3-kinase; TGF-β1, transforming growth factor β-1; TILs, tumor-infiltrating lymphocytes; TIM-3, T cell immunoglobulin and mucin domain-containing protein-3; TME, tumor microenvironment; Treg, regulatory T cell; UV, ultraviolet. Relevant references are shown in parentheses.
